# Substrate-specific regulation of the mTORC1 pathway by G protein-coupled receptors

**DOI:** 10.1042/BCJ20260165

**Published:** 2026-09-03

**Authors:** Samuel J. Atkinson, Florentina Negoita, Yuichiro Ioi, William V. Ritchie, Kyle Thompson, Max Gardner, Peter T. Ashdown, Kristina Hellberg, Terunao Takahara, Kei Sakamoto, Dawn Thompson, James N. Hislop, Riko Hatakeyama

**Affiliations:** 1 Institute of Medical Sciences, School of Medicine, Medical Sciences & Nutrition, University of Aberdeen, Foresterhill, Aberdeen AB25 2ZD, U.K.; 2 Novo Nordisk Foundation Center for Basic Metabolic Research, University of Copenhagen, Copenhagen, 2200, Denmark; 3 Graduate School of Bioagricultural Sciences, Nagoya University, Furo-cho, Chikusa-ku, Nagoya 464-8601, Japan

**Keywords:** G protein-coupled receptor (GPCR), mammalian/mechanistic Target of Rapamycin Complex 1 (mTORC1), muscarinic acetylcholine receptor M5 (M5R)

## Abstract

The mammalian/mechanistic Target of Rapamycin Complex 1 (mTORC1) orchestrates cell growth and metabolism in response to diverse extracellular and intracellular cues. mTORC1 phosphorylates a broad range of substrates, each of which plays important physiological roles. Emerging evidence suggests that mTORC1 can respond to upstream signals in a nuanced manner, enabling differential regulation of individual substrates and, consequently, specific downstream biological processes. Phosphorylation of non-canonical mTORC1 substrates, such as the lysosome biogenesis regulator transcription factor EB (TFEB), can be regulated independently of phosphorylation of canonical substrates. However, the nature of signals that determine the signaling selectivity of mTORC1 remains incompletely understood. Here, we studied mTORC1 regulation by G protein-coupled receptors (GPCRs). We found that phosphorylation of TFEB responds to GPCRs differently compared with canonical mTORC1 substrates controlling protein synthesis, such as S6K1 and 4EBP1. In particular, the muscarinic acetylcholine receptor M5 (M5R) promoted phosphorylation of S6K1 and 4EBP1 while triggering TFEB dephosphorylation. Consequently, M5R stimulated protein synthesis without inhibiting lysosome biogenesis. mTORC1 can thus separately regulate anabolic and catabolic processes under the control of M5R. The present study highlights the importance of reassessing the effects of GPCRs on mTORC1 by concurrently monitoring individual substrates, a critical consideration to be made when evaluating GPCR ligands as therapeutic agents targeting the mTORC1 pathway.

## Introduction

Cells adapt to the changing environment by fine-tuning various biological processes. At the core of the cellular adaptation mechanism is the mammalian/mechanistic Target of Rapamycin Complex 1 (mTORC1), a master regulator of cell growth and metabolism [1,2]. mTORC1 is a serine/threonine kinase that gets activated by pro-growth signals such as nutrients (e.g., amino acids) and growth factors (e.g., insulin). mTORC1, in turn, activates pro-growth cellular processes such as anabolic reactions (e.g., protein synthesis) while repressing catabolic reactions (e.g., protein degradation via autophagy) through phosphorylating its substrate proteins. mTORC1 is activated by coordinated actions of Rag and Rheb small GTPases, which mainly relay amino acids and insulin/growth factor signals, respectively. mTORC1 plays a pivotal role in cellular/organismal homeostasis in humans and is implicated in a range of conditions, including cancer, diabetes, neurodegeneration, and aging. Developing effective therapeutic strategies for these diseases requires a deeper mechanistic understanding of mTORC1 regulation.

An emerging theme in the mTORC1 research field is mTORC1’s differential signaling to individual substrates. Depending on upstream stimuli, the phosphorylation patterns of several ‘non-canonical’ mTORC1 substrates, including the transcription factor EB (TFEB), a regulator of lysosome biogenesis [3], respond differently to that of canonical mTORC1 substrates such as the ribosomal protein S6 kinase 1 (S6K1) and the eukaryotic translation initiation factor 4E-binding protein 1, regulators of global protein synthesis [4–6]. Unlike phosphorylation of canonical substrates that requires activation of both Rag and Rheb small GTPases, TFEB phosphorylation is thought to only respond to Rag GTPases.

Two models have been proposed to explain the distinct behavior of TFEB, which are not mutually exclusive. One model attributes the difference to the unique substrate recruitment mechanism, in which Rag GTPases physically interact with TFEB (but not S6K1 or 4EBP1) and present it to mTORC1 [4,7]. Another model assigns these substrates to spatially distinct mTORC1 pools; the cytosolic mTORC1 pool phosphorylates S6K1 and 4EBP1, while the lysosomal mTORC1 pool phosphorylates TFEB [6]. In either case, a comprehensive view is missing on how various external cues that are typically sensed by cell-surface receptors differentially feed into individual mTORC1 substrates.

G protein-coupled receptors (GPCRs) constitute the largest cell-surface receptor family, being encoded by more than 800 genes in the human genome [8,9]. For many years GPCR signaling was thought to occur exclusively via activation of heterotrimeric G-proteins, with distinct Gα subtypes triggering distinct downstream events. G_q_ stimulates phospholipase C; G_s_ and G_i_ stimulate and inhibit cyclic AMP production, respectively; and G_12/13_ activates Rho small GTPases [10,11]. This original paradigm has since been challenged, with the identification of G protein-independent signaling cascades, often proposed to be mediated by β-arrestin proteins [12].

GPCRs sense diverse signals ranging from chemicals such as growth factors and odorants to physical parameters such as light. Characterizing any GPCR-mTORC1 signaling therefore has wide-reaching implications for cellular adaptation mechanisms operating in diverse contexts across the human body. Moreover, GPCRs are highly druggable proteins, as more than 30% of FDA-approved drugs target GPCRs [13]. To harness the therapeutic potential of these drugs and enable their safe and effective repurposing across diverse diseases, it is essential to elucidate how GPCRs transmit signals to downstream pathways such as the mTORC1 pathway.

Substantial efforts have been made to elucidate how GPCRs modulate mTORC1 signaling ([14]; reviewed in [15]), with some GPCRs shown to activate, while others inhibit, mTORC1. A largely overlooked aspect, however, is the substrate specificity of GPCR-mTORC1 signaling, as most prior studies monitored phosphorylation of only canonical mTORC1 substrates such as S6K1 and/or 4EBP1. In the present study, we examined in parallel the response of S6K1, 4EBP1, and the non-canonical mTORC1 substrate TFEB to GPCR stimulation. We found that TFEB responds to GPCR stimulation differently to S6K1 and 4EBP1, allowing simultaneous operation of protein synthesis and lysosome biogenesis. Our findings reveal substrate/downstream specificity as a novel layer of the GPCR-mTORC1 signaling.

## Results

### Development of M5R-DREADD cell line

We examined how phosphorylation of various mTORC1 substrates—S6K1, 4EBP1, and TFEB—responds to GPCR stimulation. As G_q_ has been previously implicated in mTORC1 signaling [15], we investigated a G_q_-coupled GPCR with the muscarinic acetylcholine receptor M5 (M5R) as a model. We chose HEK293, which expresses a broad range of G proteins [16], as the model cell line.

**Figure 1:**
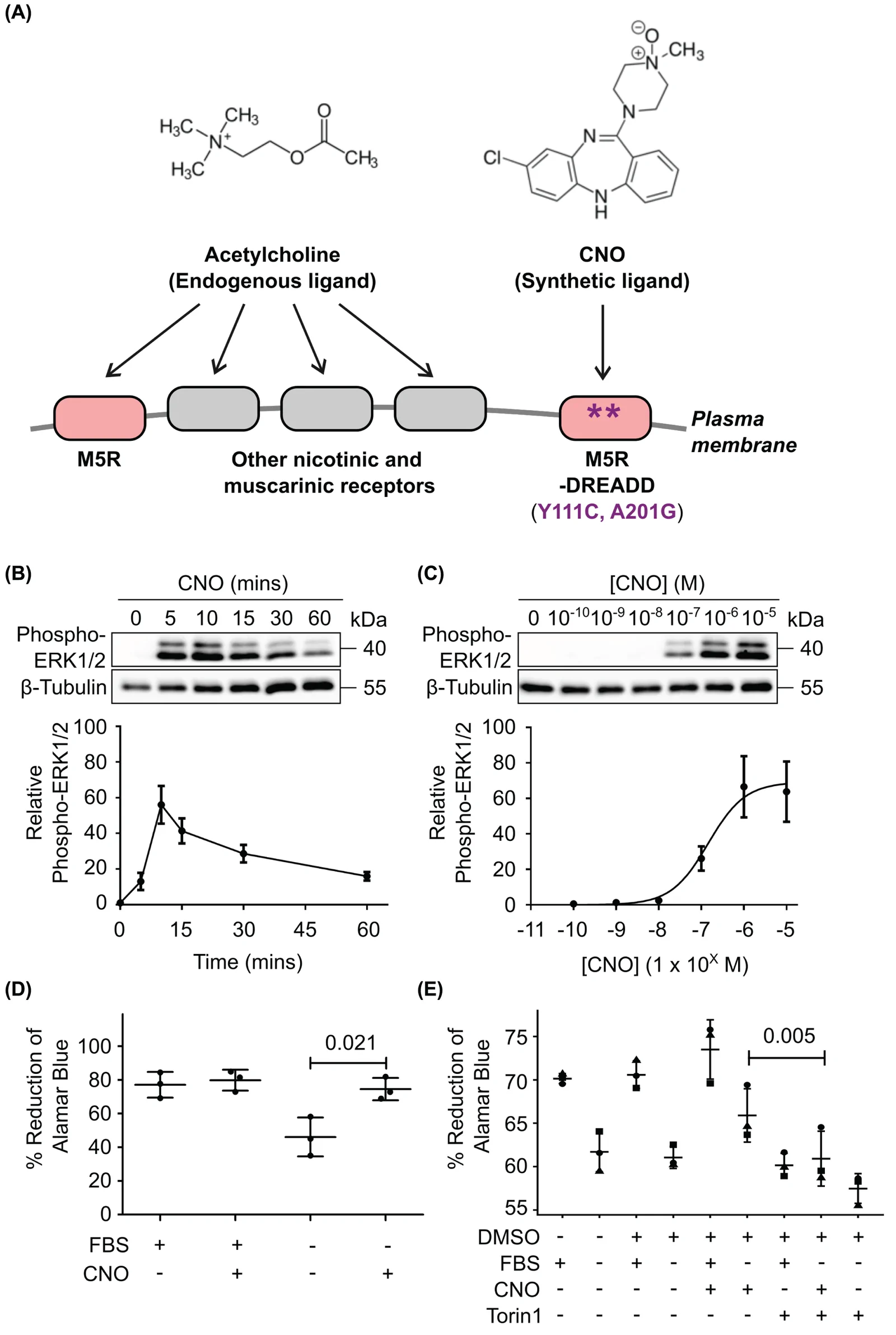
Construction and validation of the M5R-DREADD cell line (**A**) Experimental setting for M5R-specific stimulation. M5R was converted into M5R-DREADD by introducing the indicated point mutations. In cells stably expressing M5R-DREADD, the synthetic ligand CNO selectively stimulates M5R-DREADD, unlike the endogenous M5R ligand acetylcholine, which binds to nicotinic and muscarinic receptor subtypes. (**B**) HEK293 cells expressing M5R-DREADD were serum starved for 2 h prior to stimulation with 1 μM CNO for the indicated time. Cells were lysed and analyzed by SDS–PAGE and western blotting. Quantitative analysis of *n* = 3 experiments is shown at the bottom (mean ± SEM). (**C**) HEK293 cells expressing M5R-DREADD were serum starved for 2 h prior to stimulation with the indicated concentration of CNO for 7 min. Cells were lysed and analyzed by SDS–PAGE and western blotting. Quantitative analysis of *n =* 3 experiments is shown at the bottom (mean ± SEM). (**D**) Metabolic activity of HEK293 cells expressing M5R-DREADD was measured by the reduction of Alamar Blue after overnight stimulation with 1 μM of CNO with or without serum starvation. Quantitative analysis of *n* = 3 experiments (averaged from 16 technical replicates each) is shown (mean ± SD). *P*-value by unpaired Student’s *t*-test (two-tailed) is shown. (**E**) Metabolic activity of HEK293 cells expressing M5R-DREADD was measured by the reduction of Alamar Blue after overnight stimulation with 1 μM of CNO with or without serum starvation, DMSO treatment, and 100 nM Torin1 treatment. Quantitative analysis of *n* = 3 experiments (averaged from six technical replicates each) is shown (mean ± SD). Symbols represent individual experiments. *P*-value by paired Student’s *t*-test (one-tailed) is shown.

Acetylcholine, the endogenous ligand of M5R, stimulates nicotinic and muscarinic receptor subtypes, a number of which are endogenously expressed in HEK293 cells [17] (Figure 1A left). To specifically observe the effects of M5R activation, we developed a system to selectively stimulate M5R. We took advantage of the previously described mutations that make muscarinic receptors into ‘designer receptor exclusively activated by designer drugs (DREADD)’ receptors, which are uniquely activated by the synthetic ligand clozapine N-oxide (CNO) (Figure 1A right) [18].

We generated a HEK293 cell line stably expressing M5R-DREADD. The M5R-DREADD cells responded to CNO in a dose-dependent manner as expected, triggering phosphorylation of its downstream MAP kinase ERK1/2 (Figure 1B,C, showing the activation kinetics and concentration-response curve, respectively). CNO robustly enhanced the metabolic activity (reducing power) of serum-starved M5R-DREADD cells (Figure 1D), suggesting the positive role of M5R in cell growth and/or proliferation. The metabolic activity was blocked by the mTOR inhibitor Torin1 (Figure 1E). Our data established the M5R-DREADD cell line as a useful model to investigate growth/proliferation signaling, including the mTORC1 pathway.

### M5R regulates mTORC1 in a substrate-specific manner

**Figure 2:**
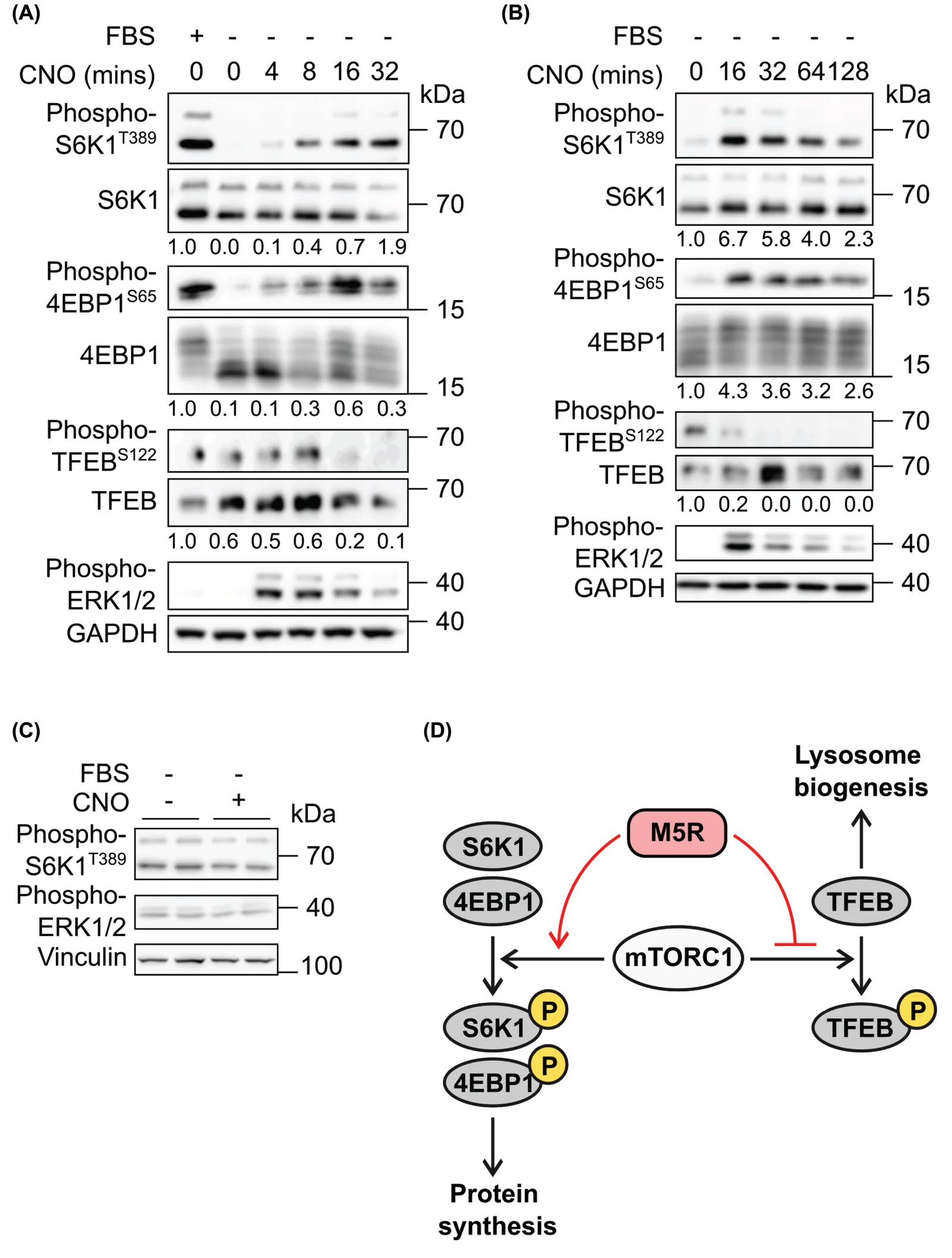
Substrate-specific regulation of mTORC1 by M5R (**A**) HEK293 cells expressing M5R-DREADD were starved of FBS or given fresh FBS-containing media for 2 h before being stimulated with 1 μM CNO for up to 32 min. Cells were lysed and analyzed by SDS–PAGE and western blotting. Numbers under blots indicate relative phosphorylation levels of each protein (*n* = 1 experiment for the entire time course; the effect of CNO has been confirmed in 2 independent, single time-point experiments). (**B**) HEK293 cells expressing M5R-DREADD were starved of FBS or given fresh FBS-containing media for 2 h before being stimulated with 1 μM CNO for up to 128 min. Cells were lysed and analyzed by SDS–PAGE and western blotting. Numbers under blots indicate relative phosphorylation levels of each protein (*n* = 1 experiment for the entire time course). (**C**) Wild-type HEK293 cells were starved of FBS or given fresh FBS-containing media for 2 h before being stimulated with 1 μM CNO for 32 min. Cells were lysed and analyzed by SDS–PAGE and western blotting (*n* = 2 experiments). (**D**) Schematic showing the proposed dualistic regulation of mTORC1 substrates by M5R. S6K1/4EBP1 phosphorylation is promoted and TFEB phosphorylation is suppressed by M5R, resulting in simultaneous activation of all three substrates.

We examined phosphorylation of canonical mTORC1 substrates, S6K1 and 4EBP1, that promote global protein synthesis. We serum-starved M5R-DREADD cells to bring the mTORC1 activity down to a basal level before stimulation with CNO. CNO treatment triggered phosphorylation of S6K1, as probed by the phospho-specific antibody (Figure 2A,B, panel 1 for a short and long time course, respectively). This effect of CNO was specifically through M5R stimulation, because it was not seen in the parental wild-type HEK293 line not expressing M5R-DREADD (Figure 2C). Phosphorylation of S6K1 coincided with that of 4EBP1 (Figure 2A,B, panel 3).

We then examined the lysosome biogenesis regulator TFEB, as its phosphorylation by mTORC1 is regulated in a non-canonical, Rheb-independent manner. Strikingly, phosphorylation of TFEB, as probed by the phospho-specific antibody, was rather down-regulated by CNO treatment after 16 min (Figure 2A,B, panel 5), suggesting inhibition of mTORC1 activity toward TFEB. These results suggest that M5R activates mTORC1 toward S6K1 and 4EBP1 while inhibiting it toward TFEB (Figure 2D).

We noticed the slow kinetics of mTORC1 substrate phosphorylation by M5R, which did not peak until 16 or 32 min in the case of S6K1 (Figure 2A,B, panel 1). This was in stark contrast to the kinetics of ERK1/2 phosphorylation that peaked at, or potentially even before, 4 min of CNO treatment (Figure 2A, panel 7). This observation raises the intriguing possibility that mTORC1 is activated only after M5R is internalized, as its endocytosis steadily progresses over 30 min upon ligand exposure [19] (see also Dyngo-4a experiments below). M5R appears to transmit qualitatively different signals over time, an early signal via ERK1/2 and a late signal via mTORC1.

### M5R stimulates protein synthesis

**Figure 3:**
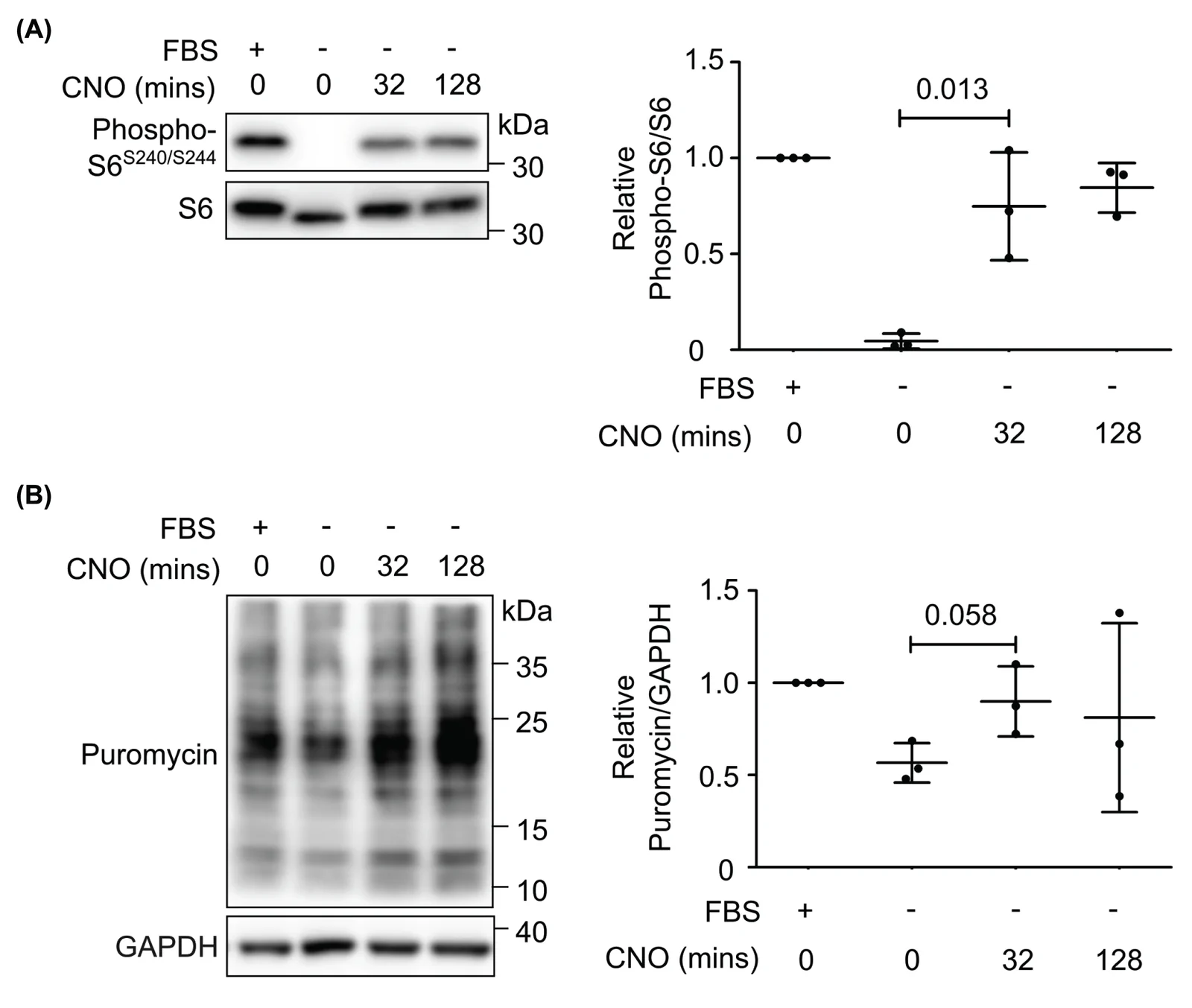
M5R stimulates protein synthesis (**A**) HEK293 cells expressing M5R-DREADD were starved of FBS or given fresh FBS-containing media for 2 h before being stimulated with 1 μM CNO for the indicated times. Cells were lysed and analyzed by SDS–PAGE and western blotting. Quantitative analysis of *n* = 3 experiments is shown on the right (mean ± SD). *P*-value by unpaired Student’s *t*-test (two-tailed) is shown. (**B**) HEK293 cells expressing M5R-DREADD were starved of FBS or given fresh FBS-containing media for 2 h before being stimulated with 1 μM CNO for the indicated times. Puromycin was added at 5 μg/ml to each condition for 15 min before lysis and analysis by SDS–PAGE and western blotting. Quantitative analysis of *n* = 3 experiments is shown on the right (mean ± SD). *P*-value by unpaired Student’s *t*-test (two-tailed) is shown.

Having observed the opposite effects of M5R activation on phosphorylation of canonical substrates (S6K1 and 4EBP1) and that of TFEB, we then examined further downstream cellular processes. Phosphorylation of S6K1 and 4EBP1 by mTORC1 promotes global protein synthesis [1,2]. S6K1 exerts this effect partly through phosphorylation of the ribosomal protein S6. As expected, CNO robustly triggered S6 phosphorylation in M5R-DREADD cells (Figure 3A).

*De novo* protein synthesis can be monitored by the established puromycin incorporation assay [20]. As shown in Figure 3B, serum starvation caused decreased incorporation of puromycin, suggestive of slowed protein synthesis. Subsequent M5R stimulation by CNO triggered increased puromycin incorporation. These results revealed the positive effect of M5R signaling on cellular protein synthesis activity, consistent with increased phosphorylation of S6K1 and 4EBP1.

### M5R does not inhibit lysosome biogenesis

M5R stimulation caused dephosphorylation of TFEB (Figure 2A,B). TFEB dephosphorylation upon amino acid starvation triggers its nuclear translocation and through activating a transcriptional program, promotes lysosome biogenesis [21]. We examined these downstream events upon M5R stimulation.

**Figure 4:**
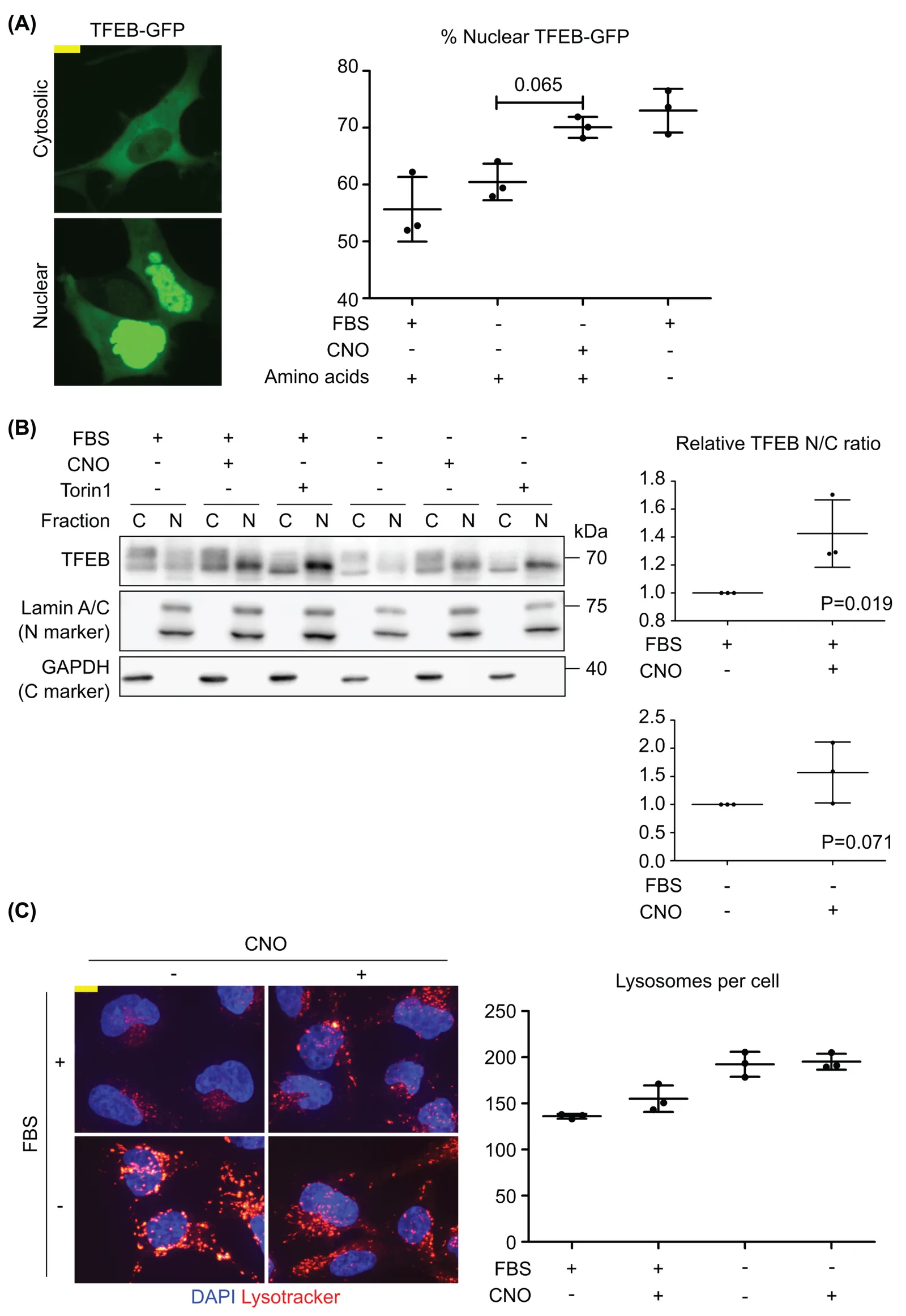
M5R does not inhibit lysosome biogenesis (**A**) HEK293 cells expressing M5R-DREADD were transfected with a TFEB-GFP-expressing plasmid. Transfected cells were starved of FBS for 2 h, except for amino acid starvation control wells, which were only starved of amino acids for a total of 45 min before imaging. Cells were stained with 2 μg/ml Hoechst 33342 and stimulated with 1 μM CNO for 32 min before imaging. Left: representative images of cells with mostly cytosolic or mostly nuclear TFEB-GFP. Quantitative analysis of *n* = 3 experiments is shown on the right (mean ± SD). Six full fields of view (containing 6 to 17 cells each) for each condition were captured per experiment. *P*-value by one-way ANOVA with the Tukey test is shown. Scale bar (yellow): 10 μm. (**B**) HEK293 cells expressing M5R-DREADD were starved of FBS or given fresh FBS-containing media for 2 h before being treated with 1 μM CNO or 100 nM Torin1 for 32 min. After cellular fractionation, cytoplasmic (C) and nuclear (N) fractions were analyzed by SDS–PAGE and western blotting. Quantitative analysis of *n* = 3 experiments is shown on the right (mean ± SD). *P*-values by unpaired Student’s *t*-test (two-tailed) are shown. (**C**) HEK293 cells expressing M5R-DREADD were starved of FBS for 2 h before stimulation with 1 μM CNO for 2 h (where indicated). Lysosomes and nuclei were stained with 20 nM LysoTracker™ Red DND-99 and 2 μg/ml Hoechst 33342, respectively, before live imaging. Quantitative analysis of *n* = 3 experiments is shown on the right (mean ± SD). Six full fields of view for each condition (containing 13 to 28 cells each) were captured per experiment. The effect of CNO was not statistically significant (one-way ANOVA with Tukey test). Scale bar (yellow): 10 μm.

As expected, CNO treatment in serum-starved M5R-DREADD cells triggered nuclear translocation of TFEB-GFP, similar to amino acid-starved cells (Figure 4A; although closely failing the statistical significance with the *P* = 0.05 cutoff). We confirmed this result by cellular fractionation of cytosol and nuclei. In both serum-fed and -starved cells, more TFEB was detected in the nuclear fraction in CNO-treated samples (Figure 4B, panel 1). The mTOR inhibitor Torin1 similarly caused nuclear translocation of TFEB, as previously reported [21].

We then quantified lysosome levels using a fluorescent lysosome-staining dye (Lysotracker-DND). As shown in Figure 4C, serum starvation increased both the intensity of Lysotracker signal and the number of the Lysotracker-positive lysosomes, suggestive of enhanced lysosome biogenesis. This trend was not reversed by subsequent M5R stimulation by CNO, in contrast with the recovery in protein synthesis (Figure 3B). The absence of a statistically significant increase in lysosome abundance suggests that M5R-induced TFEB dephosphorylation and nuclear translocation are not sufficient to promote lysosome biogenesis in HEK293 cells. To confirm this possibility, we examined the expression of TFEB target genes following treatment with the mTOR inhibitor Torin1 or the AMPK activator MK8722, both of which induce robust TFEB dephosphorylation (Supplementary Figure S1A). Consistent with our hypothesis, the expression of many TFEB target genes remained largely unchanged following either treatment (Supplementary Figure S1B), in contrast with our previous observations in mouse embryonic fibroblasts [3]. These findings indicate that, in HEK293 cells, TFEB dephosphorylation alone may be insufficient to robustly drive its transcriptional activity, highlighting the cell type-dependent nature of TFEB-mediated lysosome biogenesis and providing a mechanistic explanation for why M5R stimulation did not increase lysosome abundance in our experiments.

Taken all together, M5R activation promoted protein synthesis without suppressing lysosome biogenesis. This observation was consistent with the differential effect of M5R stimulation on phosphorylation of S6K1 and 4EBP1 and that of TFEB (Figure 2D). We conclude that M5R regulates mTORC1 in a substrate/downstream-specific manner.

### Mechanistic insights into the M5R-mTORC1 signaling

**Figure 5:**
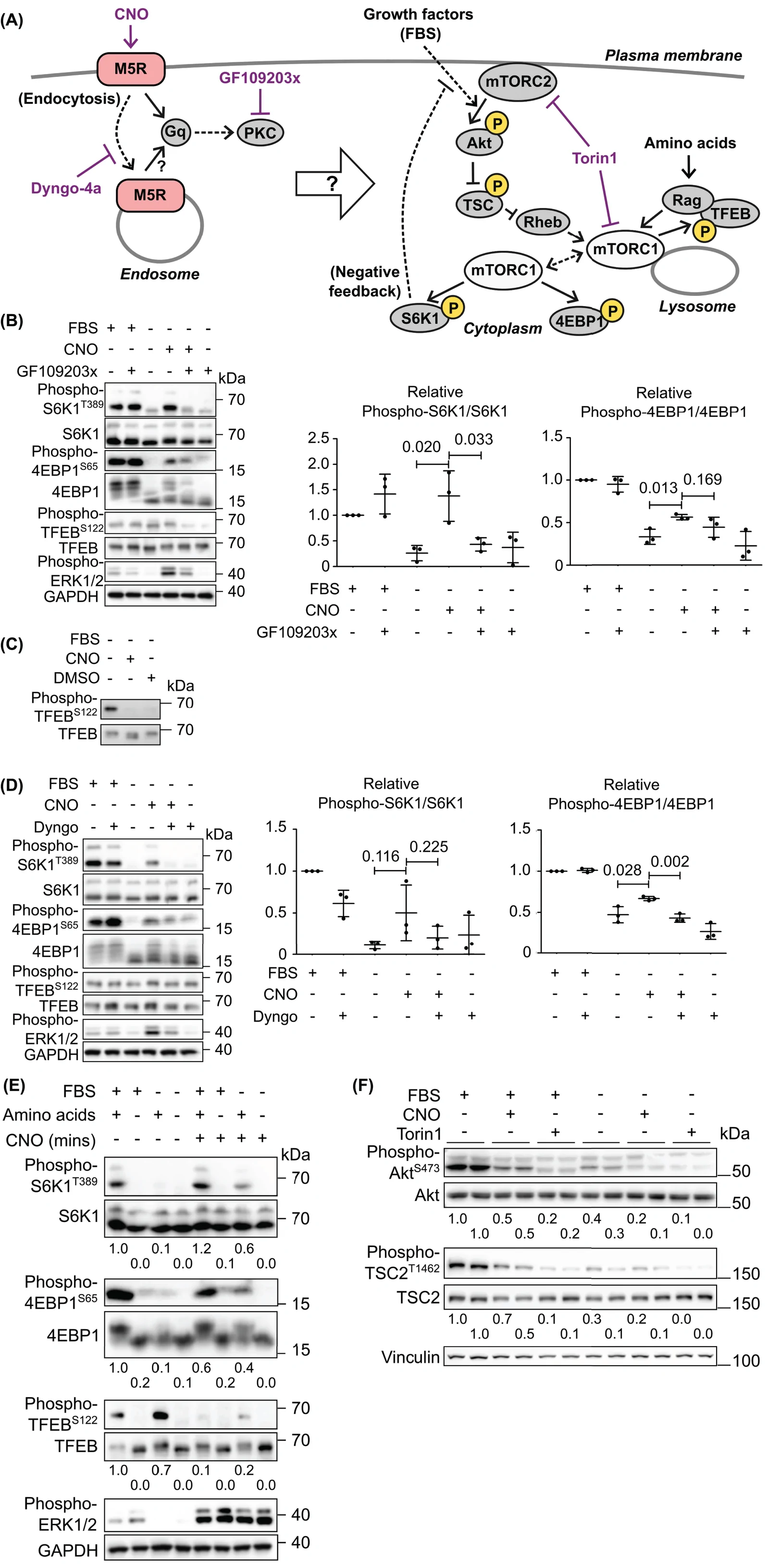
Mechanistic insights into the M5R-mTORC1 signaling (**A**) Schematic of possible outlying signaling modes of M5R to mTORC1. The points of action of CNO, GF109203x, Dyngo®-4a, and Torin1 are shown in purple. (**B**) HEK293 cells expressing M5R-DREADD were starved of FBS or given fresh FBS-containing media for 2 h before being stimulated with 1 μM CNO for 32 min. PKC was inhibited using 5 μM GF109203x for 1.5 h before CNO treatment. Cells were lysed and analyzed by SDS–PAGE and western blotting. Quantitative analysis of *n* = 3 experiments is shown on the right (mean ± SD). *P*-values by unpaired Student’s *t*-test (two-tailed) are shown. (**C**) HEK293 cells expressing M5R-DREADD were starved of FBS or given fresh FBS-containing media for 2 h before being treated with 1 μM CNO for 32 min or DMSO at 1:2000 for 1.5 h. Cells were lysed and analyzed by SDS–PAGE and western blotting (*n* = 1 experiment). (**D**) HEK293 cells expressing M5R-DREADD were starved of FBS or given fresh FBS-containing media for 2 h before being stimulated with 1 μM CNO for 32 min. The dynamin inhibitor Dyngo®-4a (to prevent endocytosis) was added at 10 μM for 30 min before CNO treatment. Cells were lysed and analyzed by SDS–PAGE and western blotting. Quantitative analysis of *n* = 3 experiments is shown on the right (mean ± SD). *P*-values by unpaired Student’s *t*-test (two-tailed) are shown. (**E**) HEK293 cells expressing M5R-DREADD were starved of FBS, amino acids, or both, or given fresh FBS-containing media for 2 h before being stimulated with 1 μM CNO for 32 min. Cells were lysed and analyzed by SDS–PAGE and western blotting. Numbers under blots indicate relative phosphorylation levels of each protein (*n* = 1 experiment). (**F**) HEK293 cells expressing M5R-DREADD were starved of FBS or given fresh FBS-containing media for 2 h before being treated with 1 μM CNO or 100 nM Torin1 for 32 min. Cells were lysed and analyzed by SDS–PAGE and western blotting. Numbers under blots indicate relative phosphorylation levels of each protein (*n* = 2 experiments).

We next addressed the mechanism of M5R-mTORC1 signaling. Because M5R activates the phospholipase C-protein kinase C (PKC) pathway downstream of G_q_ (Figure 5A, left) [10,11], we examined the effect of the PKC inhibitor GF109203x. As shown in Figure 5B, CNO-triggered phosphorylation of S6K1 and 4EBP1 in M5R-DREADD cells was blocked by GF109203x (lanes 4 and 5, panels 1 and 3), suggesting that mTORC1 activation toward S6K1 and 4EBP1 requires PKC activity.

We were unable to assess the role of PKC in the M5R-triggered dephosphorylation of TFEB, because TFEB did not respond to CNO in this experiment (lanes 3 and 4, panel 5). This was due to an unexpected effect of DMSO, which was used as the solvent of GF109203x as well as the mock control—we noticed that the DMSO treatment alone causes severe dephosphorylation of TFEB (Figure 5C). As an additional note, the effects of CNO and DMSO differed in that only the former caused a downshift of the total TFEB band (panel 2), suggesting that M5R also regulates additional posttranslational modification(s) of TFEB other than its Serine 122 phosphorylation.

It is now widely accepted that GPCRs are able to signal from both the plasma membrane and endosomes [22]. As M5R is efficiently internalized upon ligand binding [19], we asked where M5R signals to mTORC1. The remarkably slow kinetics of mTORC1’s response to CNO led us to hypothesize that M5R only signals to mTORC1 after being internalized. We addressed this possibility using the dynamin inhibitor Dyngo-4a that blocks endocytosis [19,23]. As shown in Figure 5D (lanes 4 and 5, panels 1 and 3), Dyngo-4a blocked phosphorylation of S6K1 and 4EBP1 triggered by M5R. This observation favors the post-internalization signaling model, as recently proposed for the beta-2 adrenergic receptor [24]. Our results did not inform the location where M5R signals to the mTORC1-TFEB axis because of a non-specific effect of DMSO (Figure 5C), in which Dyngo-4a was dissolved.

mTORC1 is activated by the actions of Rag and Rheb small GTPases, which mainly mediate amino acid and insulin/growth factor signals, respectively (Figure 5A, right). Notably, M5R failed to stimulate S6K1 and 4EBP1 phosphorylation in amino acid-starved cells (Figure 5E, lanes 2 and 6, panels 1 and 3; compare with serum-starved cells in lanes 3 and 7). This was not due to the failure of M5R activation, because ERK1/2 phosphorylation was similarly triggered by CNO in serum- and amino acid-starved cells (lanes 6 and 7, panel 7). Hence, M5R seems to activate the Rheb branch but not the Rag branch, the latter of which requires an amino acid signal.

Growth factors, including insulin, activate Rheb via the mTORC2-Akt cascade (Figure 5A, right). M5R does not activate this canonical upstream pathway, because CNO did not promote phosphorylation of Akt and TSC2, an Akt substrate (Figure 5F). These phosphorylation events were rather down-regulated by CNO, which could be due to the known negative feedback regulation through S6K1 (Figure 5A, right).

Altogether, the most straightforward interpretation of our data is that M5R activates the mTORC1-S6K1/4EBP1 axis through the G_q_-PKC pathway after being internalized. This signal most likely impinges on Rheb through unknown mechanisms independent from the canonical mTORC2-Akt-TSC pathway. How M5R inhibits the mTORC1-TFEB axis is less clear.

### mTORC1 regulation by natively expressed muscarinic acetylcholine receptors

**Figure 6:**
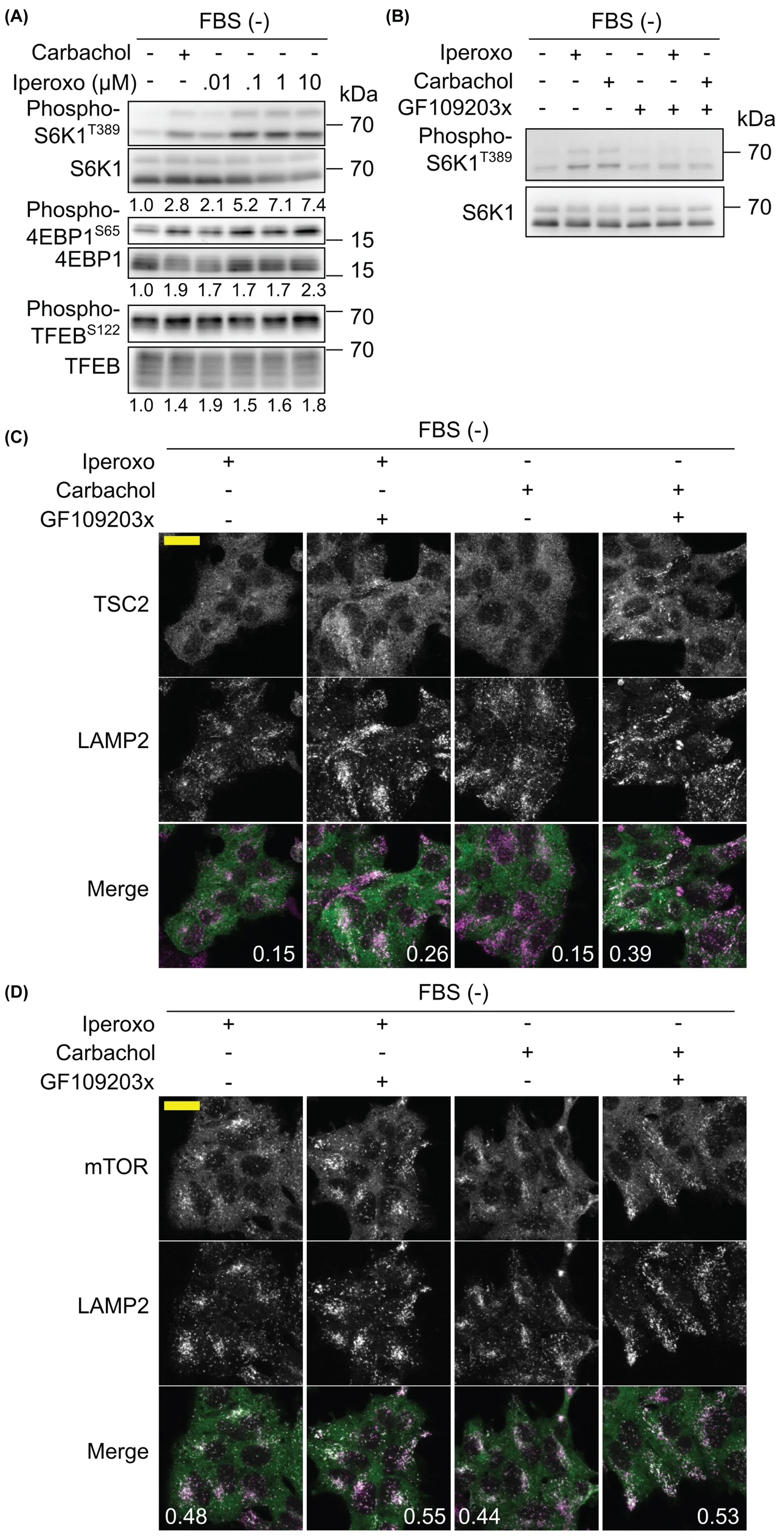
mTORC1 regulation by natively expressed muscarinic acetylcholine receptors (**A**) Wild-type HEK293 cells were starved of FBS for 3 h before being stimulated with carbachol (100 μM) or iperoxo (at the indicated concentrations) for 15 min. Cells were lysed and analyzed by SDS–PAGE and western blotting. A representative blot from *n* = 2 experiments is shown. Numbers indicate relative phosphorylation levels of each protein. (**B**) Wild-type HEK293 cells were starved of FBS for 3 h, treated with 5 μM GF109203x for 30 min, and stimulated with 100 nM iperoxo or 100 μM carbachol for 15 min. Cells were lysed and analyzed by SDS–PAGE and western blotting (*n* = 1 experiment). (**C, D**) Wild-type HEK293 cells were starved of FBS for 3 h, treated with 5 μM GF109203x for 30 min, and stimulated with 100 nM iperoxo or 100 μM carbachol for 15 min. Cells were fixed and immunostained with the indicated antibodies (*n* = 1 experiment). Merged images of TSC2 (green in C) or mTOR (green in D) and LAMP2 (magenta) are also shown. Pearson’s correlation coefficients are shown at the bottom. Scale bar (yellow): 20 μm.

To gain broader insight, we extended the analysis to GPCRs other than M5R. We first examined muscarinic acetylcholine receptors natively expressed in HEK293 cells. Wild-type HEK293 cells were serum starved and stimulated with carbachol, an acetylcholine receptor agonist, or iperoxo, a superagonist of muscarinic acetylcholine receptors [25]. As shown in Figure 6A, both carbachol and iperoxo triggered phosphorylation of S6K1 and 4EBP1 in 15 min (panels 1 and 3). The effect of carbachol and iperoxo differed from M5R activation in that they did not trigger dephosphorylation of TFEB. This could be due to the difference in receptor type and/or expression level, as the muscarinic receptor primarily expressed in HEK293 cells is M3R [26]. Other muscarinic receptors may have contributed as well.

Mechanistically, mTORC1 activation by endogenous muscarinic receptors was PKC-dependent (Figure 6B), consistent with our observations using M5R-DREADD (Figure 5B). We have previously shown that carbachol induces dissociation of TSC2 from lysosomes through the Ca^2+^-calmodulin pathway [27]. Our current findings indicate that PKC also contributes to TSC2 relocalization, as PKC inhibition enhanced lysosomal localization of TSC2 in cells treated with carbachol or iperoxo (Figure 6C). This regulation might be mediated through direct phosphorylation of TSC2 by PKC, as previously reported [28]. In contrast, mTOR remained localized to lysosomes regardless of treatment with carbachol, iperoxo, or the PKC inhibitor GF109203x (Figure 6D). Together, these findings suggest that endogenous muscarinic receptors activate mTORC1 toward canonical substrates, including S6K1 and 4EBP1, at least in part by promoting the displacement of TSC2 from lysosomes through activation of the PKC and Ca^2+^-calmodulin pathways.

### mTORC1 regulation by other GPCRs

**Figure 7:**
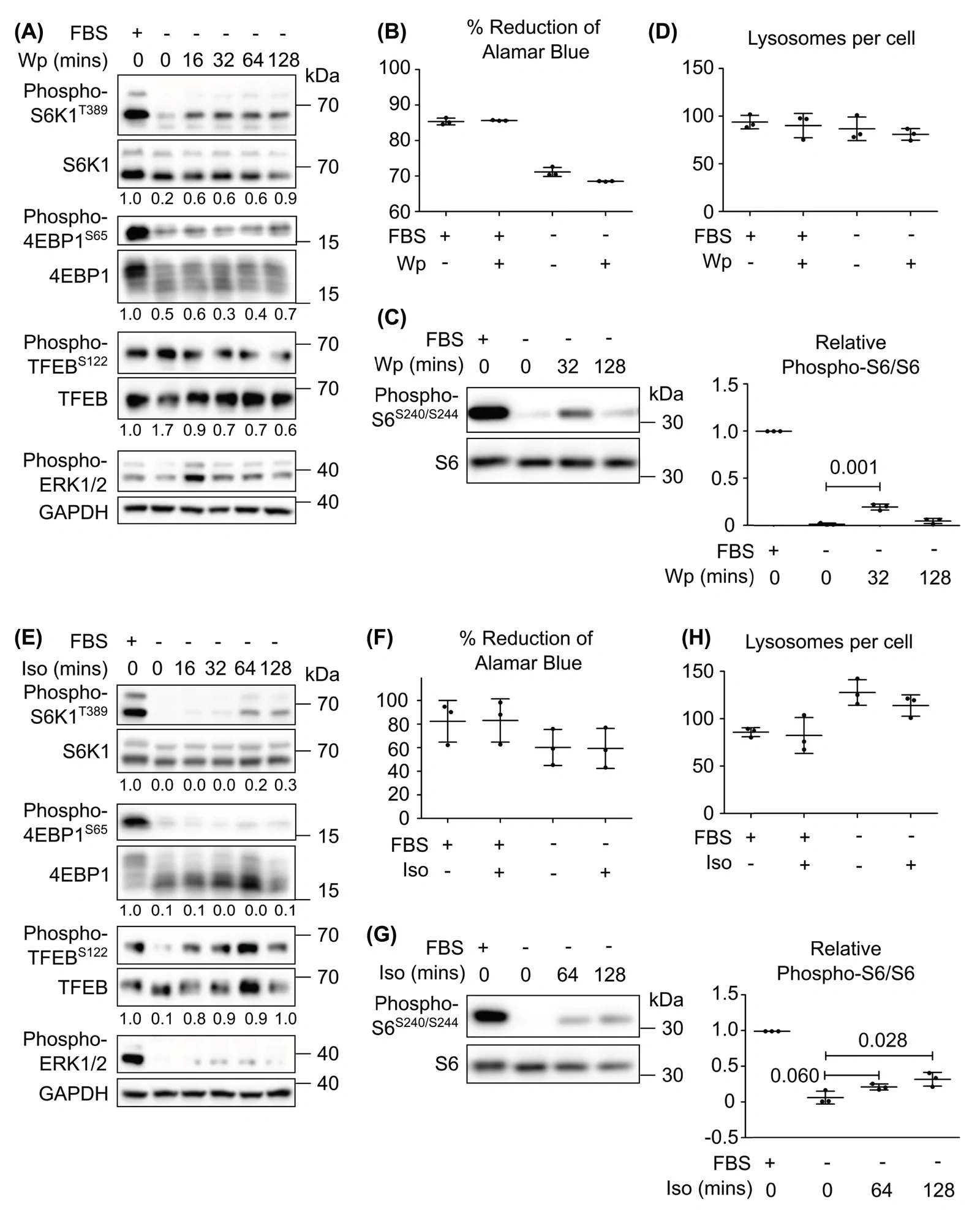
Differential response of mTORC1 substrates to other GPCRs (**A**) HEK293 cells expressing FPR2 were starved of FBS or given fresh FBS-containing media for 2 h before being stimulated with 1 μM WKYMVm peptide (Wp) for up to 128 min. Cells were lysed and analyzed by SDS–PAGE and western blotting. Numbers under blots indicate relative phosphorylation levels of each protein (*n* = 1 experiment for the entire time course; the effect of WKYMVm has been confirmed in seven independent, single time-point experiments). (**B**) Metabolic activity of HEK293 cells expressing FPR2 was measured by the reduction of Alamar Blue after overnight stimulation with 1 μM WKYMVm peptide with or without serum starvation. Quantitative analysis of *n* = 3 experiments (averaged from 16 technical replicates each) is shown (mean ± SD). (**C**) HEK293 cells expressing FPR2 were starved of FBS or given fresh FBS-containing media for 2 h before being stimulated with 1 μM WKYMVm peptide for the indicated times. Cells were lysed and analyzed by SDS–PAGE and western blotting. Quantitative analysis of *n* = 3 experiments is shown on the right (mean ± SD). *P*-value by unpaired Student’s *t*-test (two-tailed) is shown. (**D**) HEK293 cells expressing FPR2 were starved of FBS for 2 h before stimulation with 1 μM WKYMVm peptide for 2 h (where indicated). Lysosomes and nuclei were stained with 20 nM LysoTracker™ Red DND-99 and Hoechst 33342 before live imaging. The result of a quantitative analysis of *n* = 3 experiments is shown (mean ± SD). (**E**) Wild-type HEK293 cells were starved of FBS or given fresh FBS-containing media for 2 h before being stimulated with 1 μM isoprenaline (Iso) for up to 128 min. Cells were lysed and analyzed by SDS–PAGE and western blotting. Numbers under blots indicate relative phosphorylation levels of each protein (*n* = 1 experiment for the entire time-course; the effect of isoprenaline has been confirmed in 8 independent, single time-point experiments). (**F**) Metabolic activity of wild type HEK293 cells was measured by the reduction of Alamar Blue after overnight stimulation with 1 μM isoprenaline with or without serum starvation. Quantitative analysis of *n* = 3 experiments (averaged from 16 technical replicates each) is shown (mean ± SD). (**G**) Wild-type HEK293 cells were starved of FBS or given fresh FBS-containing media for 2 h before being stimulated with 1 μM isoprenaline for the indicated times. Cells were lysed and analyzed by SDS–PAGE and western blotting. Quantitative analysis of *n* = 3 experiments is shown on the right (mean ± SD). *P*-values by unpaired Student’s *t*-test (two-tailed) are shown. (**H**) Wild-type HEK293 cells were starved of FBS for 2 h before stimulation with 1 μM isoprenaline for 2 h (where indicated). Lysosomes and nuclei were stained with 20 nM LysoTracker™ Red DND-99 and Hoechst 33342 before live imaging. The result of a quantitative analysis of *n* = 3 experiments is shown (mean ± SD).

We then asked whether substrate-specific regulation of mTORC1 is unique to muscarinic receptors or G_q_-coupled GPCRs. We examined the G_i_-coupled formyl peptide receptor 2 (FPR2). FPR2-expressing HEK293 cells previously generated [29] were serum starved and treated with the peptide ligand WKYMVm, a potent FPR2 agonist [30]. As shown in Figure 7A, WKYMVm triggered phosphorylation of S6K1 and 4EBP1 in 16 min (panels 1 and 3; see also upshift of the total 4EBP1 bands in panel 4), though not affecting the cellular metabolic activity (Figure 7B). S6 phosphorylation was promoted as expected (Figure 7C). FPR2 weakly inhibited TFEB phosphorylation (Figure 7A, panel 5), but lysosome biogenesis was unaffected (Figure 7D). Interestingly, serum starvation did not significantly affect the lysosome level in this cell line, which could be due to the WKYMVm-independent constitutive activity of FPR2.

We then examined G_s_-coupled GPCRs. We treated wild-type HEK293 cells, which endogenously express the G_s_-coupled beta-2 and -1 adrenergic receptors (β2AR and β1AR), with their ligand isoprenaline [31]. Isoprenaline triggered phosphorylation of S6K1 in 64 min (Figure 7E, panel 1) but did not promote phosphorylation of 4EBP1 (panel 3). Isoprenaline did not affect cellular metabolic activity (Figure 7F), despite increased S6 phosphorylation (Figure 7G). In contrast with the response to M5R activation, TFEB phosphorylation was promoted by isoprenaline stimulation (Figure 7E, panel 5), with lysosome biogenesis unaffected (Figure 7H).

To summarize, M5R, FPR2, and β2AR/β1AR all activated mTORC1 toward S6K1. M5R and FPR2 also activated the mTORC1-4EBP1 branch. However, M5R strongly inhibited mTORC1 toward TFEB, uniquely among the three GPCRs. Hence, different GPCRs regulate mTORC1 with varied substrate specificities.

## Discussion

The present study uncovered the substrate/downstream-specific mode of mTORC1 regulation by GPCRs, revealing an additional layer of complexity to this signaling axis. Notably, the response of TFEB was very different (even opposite for M5R) from that of canonical mTORC1 substrates, S6K1 and 4EBP1. With the limited number of GPCRs tested in the present study, we do not rule out the presence of GPCRs that globally regulate mTORC1 without substrate specificity. Regardless, a lesson to be drawn from this is that we need to monitor multiple mTORC1 substrates, including TFEB, when assessing the effects of GPCRs. This perspective has been largely overlooked in previous studies, which have predominantly focused on canonical mTORC1 targets and, consequently, described GPCRs as general activators or inactivators of mTORC1 [15].

Further study is required to elucidate the signaling pathways through which individual GPCRs regulate mTORC1. Although our pharmacological experiments suggested the contribution of PKC and endocytosis to M5R-dependent mTORC1 activation toward S6K1 and 4EBP1, their roles in TFEB phosphorylation remain inconclusive. This is because the vehicle control (DMSO) alone induced TFEB dephosphorylation, thereby confounding the interpretation of the inhibitor experiments (Figure 5C). Further studies employing complementary genetic approaches will be important to validate and extend these pharmacological findings.

Mechanistically, the unique response of TFEB upon GPCR stimulation can be partly explained if GPCRs signal to mTORC1 through the Rheb branch, which TFEB is uniquely unresponsive to. This interpretation aligns with our observation that M5R stimulation overrides FBS starvation but not amino acid starvation (Figure 5E). It is indeed known that the MEK-ERK1/2 cascade, which acts downstream of many GPCRs, including the ones examined here, activates Rheb by inhibiting its negative regulator TSC complex [32]. Regulation of TSC2 localization through the PKC and Ca^2+^-calmodulin pathways (Figure 6C) [27] appears to serve as an additional signaling mechanism for G_q_-coupled GPCRs. Another factor could be the subcellular location of phosphorylation events. Given that TFEB is phosphorylated at lysosomes rather than the cytoplasm as proposed for S6K1 and 4EBP1 [6], GPCRs may exert substrate-specific effects by modulating mTORC1 activity in a location-specific manner. The involvement of phosphatases antagonizing mTORC1 should also be considered, which could be different for each mTORC1 substrate [5].

The non-canonical response of TFEB observed in this and other studies indicates that a catabolic process (lysosome biogenesis) and an anabolic process (protein synthesis) can be separately regulated, even though both are governed by mTORC1. A similar decoupling of catabolic and anabolic processes is observed in the budding yeast model, in which protein synthesis and autophagy are regulated by spatially distinct TORC1 pools [33]. This may look counterintuitive from the traditional view in the TORC1 field that activation of anabolism or catabolism should coincide with inhibition of the other. Such coupling would make sense in the context of nutrient response, where cells adapt their bulk mass to nutrient availability. However, the situation is different when cells respond to other stimuli such as growth factors or stresses. Cells would need to change themselves qualitatively by remodeling the proteome, not necessarily changing their total mass. For that, they would need to synthesize proteins that are needed in the new environment and simultaneously degrade proteins not needed anymore. The latter catabolic process and subsequent recycling of resources (e.g., amino acids) fuel the former anabolic processes. In such a situation, it would be beneficial to activate both anabolism and catabolism by decoupling their regulations.

There is another potential benefit of metabolic decoupling in light of cell proliferation. M5R robustly stimulated cellular metabolic activity (Figure 1D). Under this condition where cells actively proliferate, it would be beneficial to keep TFEB dephosphorylated and generate lysosomes needed for daughter cells. TFEB dephosphorylation was indeed not observed upon FPR2 or β2AR/β1AR activation, where cellular metabolic activity was unaffected (Figure 7). These benefits are, however, speculative and need to be validated by future studies in more physiological experimental settings.

The supreme druggability of GPCRs and their tissue/context-specific expression patterns make GPCR ligands attractive therapeutic tools to manipulate the mTORC1 pathway to treat cancer, diabetes, neurodegeneration, and aging. The substrate/downstream specificity revealed in this study further enhances the potential of GPCR ligands, as it theoretically enables selective manipulation of the desired downstream functions of mTORC1, which should be more effective and safer than pan-mTORC1 manipulation. For example, downstream-specific targeting could be beneficial for treating triple-negative breast cancer, of which invasion is induced by mTORC1 inhibition toward TFEB [34]. A potential solution in such a case is to inhibit mTORC1 toward canonical substrates (to block cancer cell growth) but not toward TFEB (to avoid inducing invasion), leveraging the substrate-specific effect of GPCR-targeting drugs. This translational potential warrants a comprehensive investigation of the mTORC1 substrate specificity for a wider range of GPCRs.

## Methods

### Antibodies and reagents

For western blotting, all antibodies were purchased from Cell Signaling Technologies except anti-GAPDH (Proteintech, 60004-1-Ig) and anti-puromycin (Merck, MABE343). Anti-S6K (2708), anti-phospho-S6K Thr389 (9205 or 9234), anti-4EBP1 (9644), anti-phospho-4EBP1 Ser65 (13443), anti-TFEB (4240), anti-phospho-TFEB Ser122 (87932), anti-S6 (2217), anti-phospho-S6 Ser240/Ser244 (5364) anti-phospho-p44/42 MAPK (Erk1/2) Thr202/Tyr204 (4370), anti-Vinculin (13901), anti-Lamin A/C (4777), anti-Akt (4691), anti-phospho-Akt Ser473 (4060), anti-TSC2 (4308), and anti-phospho-TSC2 Thr1462 (3617) were used at 1:1000. Anti-GAPDH was used at 1:30,000. For immunostaining, anti-mTOR (7C10, 1:400 dilution), anti-TSC2 (D93F12, 1:1000), anti-LAMP2 (H4B4, Proteintech 65054-1-Ig, 1:500), Alexa Fluor 488 donkey anti-mouse IgG (Invitrogen, 1:1000), and Alexa Fluor 555 donkey anti-rabbit IgG (Invitrogen, 1:1000) were used. Unless otherwise stated, common reagents were purchased from Thermo Fisher Scientific.

### Cell culture

All cell lines (HEK293 and derivatives) were cultured in DMEM high glucose (Merck, D5671) supplemented with 1 mM sodium pyruvate (Thermo Fisher Scientific, 11360-039), 1% glutamine (Merck, G7513), and 10% FBS (Biosera, 10011500) at 37°C in an atmosphere of 5% CO_2_. Cell lines were regularly tested for the presence of mycoplasma contamination. FPR2 stably expressing cells were previously generated as described in [29]. DMEM without amino acids (VWR, USBID9800-13) was supplemented with glucose to match DMEM high-glucose above, 1 mM sodium pyruvate, and 10% dialysed FBS (lacking amino acids). For dialysing FBS, the same FBS batch used for all experiments was dialysed against PBS twice overnight at 4°C using a 3500 Da cutoff Snakeskin™ dialysis membrane (Thermo Fisher Scientific, 88242).

### Muscarinic acetylcholine receptor M5 DREADD stable cell line

HEK293 cells were from the ATCC. The M5 DREADD receptor was generated based on the M3R DREADD [35]. Briefly, M5R cDNA was obtained from cDNA.org and subcloned into the Flag pcDNA3.1+ zeo [29]. Site-directed mutagenesis (QuikChange, Agilent) was then used to introduce the mutations in the third and fifth transmembrane domains (Y111C and A201G) to confer sensitivity to CNO. Stables were generated by selection with zeocin and identified by anti-Flag immunofluorescence determined by microscopy.

### GPCR stimulation

Unless specified otherwise, all cell lines were washed with PBS and then starved of amino acids or FBS for 2 h before treatment with the GPCR agonists at 1 μM: CNO (Tocris/Bio-Techne, 6329), Isoprenaline (Sigma/Merck, I6504), or WKYMVm (Tocris/BioTechne, 1800) in M5R-DREADD, wild-type, or FPR2-expressing HEK293 cells, respectively, for the indicated timepoints. Natively expressed muscarinic acetylcholine receptors were stimulated by carbachol (Santa Cruz, sc-202092) or iperoxo (Cayman Chemical, 27235). For inhibitor experiments, 10 μM Dyngo®-4a (Abcam, ab120689) or 5 μM PKC inhibitor GF109203x (Sigma/Merck, B6292) was added to the culture media 30 min or 1.5 h, respectively, before GPCR stimulation. One hundred nanomolar of Torin1 was applied for 32 min.

### Global translation assay

Cells were stimulated as above, and puromycin (Thermo Fisher Scientific, 227420500) was added 15 min before lysis at 5 μg/ml as per the SUNSET assay protocol [20,36].

### Cellular fractionation

Cellular fractionation was performed with the NE-PER™ Nuclear and Cytoplasmic Extraction Reagents kit (Thermo Fisher Scientific, 78835). The cells were seeded in six-well plates at a cell density of 4 × 10^5^ cells/well. The next day they were treated as specified in the figure legends. After treatment, the cells were washed once with PBS (Substrate and Sterile Center, University of Copenhagen). The washed cells were lysed with the cytosolic extraction reagents I and II (CERI and CERII) provided in the kit to induce disruption of the cell membranes and the release of cytosolic proteins. After being scraped in cold CERI buffer containing protease and phosphatase inhibitors (500 μM phenylmethylsulfonyl fluoride [Sigma, P7626], 1 mM benzamidine hydrochloride [Sigma, 199001], 1 μg/ml leupeptin [Sigma, L2884], 1 μg/ml pepstatin A [Sigma, P5318], 1 μM microcystin-LR [Enzo Life Sciences, ALX-350-012], 1 mM sodium orthovanadate [Sigma, S6508]), the cells in CERI buffer were dispersed by brief vortexing, incubated on ice for 10 min and then mixed with CERII buffer, and further incubated on ice for 1 min. The separation of cytosolic proteins from the nuclear pellet was performed by centrifugation at 16,000×***g*** for 5 min at 4°C. The supernatant containing the cytosolic protein fraction was collected in a new tube and denatured at 100°C for 5 min in Laemmli sample buffer (50 mM Tris, pH 6.8, 2% sodium dodecyl sulfate [Sigma, 74255], 0.5 mM EDTA [Sigma, E5134], 10% glycerol [Sigma, G5516], 0.01% bromophenol blue [Sigma, 114391]). The pellet containing the nuclei was washed three times with cold PBS. Nuclear protein fractions were obtained by extraction from the nuclear pellet with Laemmli sample buffer at 100°C for 7 min. The denatured cytosolic and nuclear protein fractions were stored at −20°C until Western blot analysis.

### Western blotting

Cells were washed with PBS before being lysed directly in high SDS lysis buffer (3% SDS, 60 mM sucrose, 65 mM Tris–HCl, pH 6.8), denatured at 95°C for 5 min, homogenized by sonication, and cleared by centrifugation at 18,000×***g*** for 30 min. Lysates were quantified by a linearized Bradford assay (Abcam, ab119216) using a 595/450 nm ratio.

Twenty-five micrograms of proteins were separated in SDS–PAGE gels before being transferred to nitrocellulose membranes (VWR, 10600002). After ponceau staining, membranes were blocked with 5% milk/TBST (for anti-TFEB, phospho-TFEB, GAPDH, and phospho-MAPK antibodies), 3% milk/TBST (for anti-Vinculin, Lamin A/C, Akt, phospho-Akt, TSC2, and phospho-TSC2 antibodies) or 5% BSA/TBST (for anti-S6K, phospho-S6K, and 4EBP1 antibodies) and probed with antibodies overnight at 4°C in 5% BSA/TBST (for anti-S6K, phospho-S6K, 4EBP1, TFEB, phospho-TFEB, and phospho-MAPK antibodies), 3% milk/TBST (for anti-Vinculin, Lamin A/C, Akt, phospho-Akt, TSC2, and phospho-TSC2 antibodies) or 5% milk/TBST (for anti-GAPDH antibody). Membranes were visualized after secondary antibody incubation (Cell Signaling Technologies, 7074 and 7076) and washing with TBST using ECL substrate (Thermo Fisher Scientific, 12994780). Membranes were imaged in a Gbox gel/blot imaging system (Syngene), and densitometry analysis was performed using ImageJ [37].

### TFEB-GFP microscopy

HEK293 M5R-DREADD cells were transfected overnight using Fugene® HD (Promega, E2311) with TFEB-GFP (MRC PPU Reagents and Services, DU67822). Transfected cells were re-seeded onto eight-well chamber slides (Thistle Scientific, IB-80826) and allowed to recover overnight. Wells were washed with PBS and starved of FBS for 2 h except for amino acid starvation control wells, which were only starved of amino acids for a total of 45 min before imaging. Cells were stained with 2 μg/ml Hoechst 33342 (Thermo Fisher Scientific, 62249) and stimulated with 1 μM CNO for 32 min before imaging. Live cell images were acquired on an Ultraview VoX spinning disk confocal microscope (PerkinElmer) with a 40× objective. Six full fields of view (containing 6 to 17 cells each) for each condition were captured per experiment, and the percentage of the total TFEB-GFP signal that was nuclear was then measured. Images were analyzed with ImageJ, defining GFP signals overlapping with Hoechst signals as nuclear TFEB-GFP.

### Lysosome microscopy

Cells were seeded overnight onto eight-well chamber slides and allowed to recover overnight. The next day cells were washed with PBS and starved of FBS for 2 h, followed by 2 h of GPCR stimulation (as above). Twenty nanomolar LysoTracker™ Red DND-99 (ThermoFisher Scientific, L7528) and 2 μg/ml Hoechst 33342 were added to the wells for 30 min before imaging. Live images were acquired on an Ultraview VoX spinning disk confocal microscope (PerkinElmer) with a 63× objective.

Six full fields of view for each condition (containing 13 to 28 cells each) were captured per experiment. The Analyze Particles function in ImageJ, in combination with mild thresholding, was used to create a mask to exclude areas without cells. The Find Maxima function in ImageJ was then used to count lysosomes within the masked areas only, which was normalized to the number of cells obtained by counting Hoechst 33342-stained nuclei.

### Alamar blue assay

Cells were seeded overnight in 96-well plates before being washed with PBS and treated with DMEM +/− FBS and +/− GPCR agonist (see above) for 18 h. Media was then replaced with DMEM + 10% Alamar blue solution (Bio-Rad BUF012A) for 3 h before absorbance at 570 and 600 nm was measured on a Clariostar Plus plate reader (BMG Labtech). Analysis was performed as per the manufacturer’s recommendations.

### Immunofluorescence

Cells were seeded into 35-mm dishes containing PLL-treated glass coverslips. After 24 h, cells were washed once with serum-free DMEM and incubated in serum-free DMEM for 3 h. Cells were treated with 5 μM GF109203x for 30 min and stimulated with 100 nM iperoxo or 100 μM carbachol for 15 min. Cells were then washed once with PBS(−) and fixed with 4% paraformaldehyde in PBS(−) for 15 min, followed by washing twice with PBS(−). Cells were permeabilized with 0.1% Triton X-100/PBS(−) for 5 min, followed by washing twice with PBS(−). After blocking with 0.25% bovine serum albumin (BSA)/PBS(−) for 1 h, cells were incubated with primary antibodies at 4°C for 18 h and washed five times with 0.25% BSA/PBS(−). Cells were then incubated with secondary antibodies at room temperature for 1 h in the dark and then washed five times with 0.25% BSA/PBS(−), stained with 5 μM Hoechst 33342 for 10 min in the dark, and washed twice with 0.25% BSA/PBS(−). Cover glasses were soaked in water before mounting. Images were acquired under an FV3000 confocal laser-scanning microscope equipped with a numerical aperture oil-immersion objective (UPLXAPO60XO).

### qPCR

Quantitative RT-PCR was performed as described previously [3] with some modifications. After 6 h treatment with MK8722 (Glixx Laboratories Inc, GLXC-11445) or Torin1 (Selleck Chemicals, S2827), cells were washed once with PBS (Substrate and Sterile Center, University of Copenhagen) and lysed with Buffer RLT (QIAGEN, 79216) supplemented with 2-mercaptoethanol (Sigma, M6250; 1:100 dilution). RNA was isolated, and on-column treated with RNAse-Free DNase (QIAGEN, 79256), using the RNEasy mini kit (QIAGEN, 74106) according to the manufacturer’s instructions. RNA concentration and quality were analyzed by a NanoDrop One Spectrophotometer (ThermoFisher Scientific, Wilmington, U.S.A.). Two micrograms of RNA was reverse transcribed using Superscript III cDNA synthesis kit (Invitrogen, 18080), dNTPs (Invitrogen, 10297-018), RNaseOUT (Invitrogen, 10777019), random primers (Roche, 11034731001) and nuclease-free water (Ambion, AM9937) in a 20-μl reaction as per the manufacturer’s instructions. cDNA was diluted 20×, and 2 μl was used for qPCR reactions (10 μl total) using indicated primers (Supplementary Table S1) and PrecisionPLUS qPCR Master Mix with SYBRgreen (Primer Design, Z_PPLUS-SY-20 ml) on a LightCycler480 (Roche Diagnostics A/S, Denmark). For each experiment, cDNA was diluted to generate a standard curve to analyze primer efficiency. The delta delta Ct method was used to calculate gene expression, and data was normalized to the mean of Hprt, RNA 18S, and Tbp.

### Statistical analysis

All statistical analyses and graphs were produced in GraphPad Prism. Technical replicates were averaged for each biological replicate. Control and experimental conditions’ biological repeats were compared using unpaired Student’s *t-*tests or one-way ANOVA with Tukey tests. *P*-values are reported on graphs.

## Supplementary Material

Supplementary Figure S1 and Table S1

## Data Availability

The data are available from the corresponding author upon reasonable request.

## Open Access

Open access for this article was enabled by the participation of the University of Aberdeen in an all-inclusive Read & Publish agreement with Portland Press and the Biochemical Society under an agreement with JISC.

## Abbreviations

β2AR: beta-2 adrenergic receptor
CNO: clozapine N-oxide
DREADD: designer receptor exclusively activated by designer drugs
FPR2: formyl peptide receptor 2
GPCR: G protein-coupled receptor
M5R: muscarinic acetylcholine receptor M5
mTORC1: mammalian/mechanistic Target of Rapamycin Complex 1
PKC: protein kinase C
S6K1: S6 kinase 1
TFEB: transcription factor EB
