# Supplementary material for: Substrate-specific regulation of the mTORC1 pathway by G protein-coupled receptors
Source: Biochem J. 2026 Sep 3;483(10):1817–34. doi: 10.1042/BCJ20260165 (PMC13543022; doi:10.1042/BCJ20260165)
Supplement: Supplementary file 1 [file BCJ-2026-0165_supp.pdf]

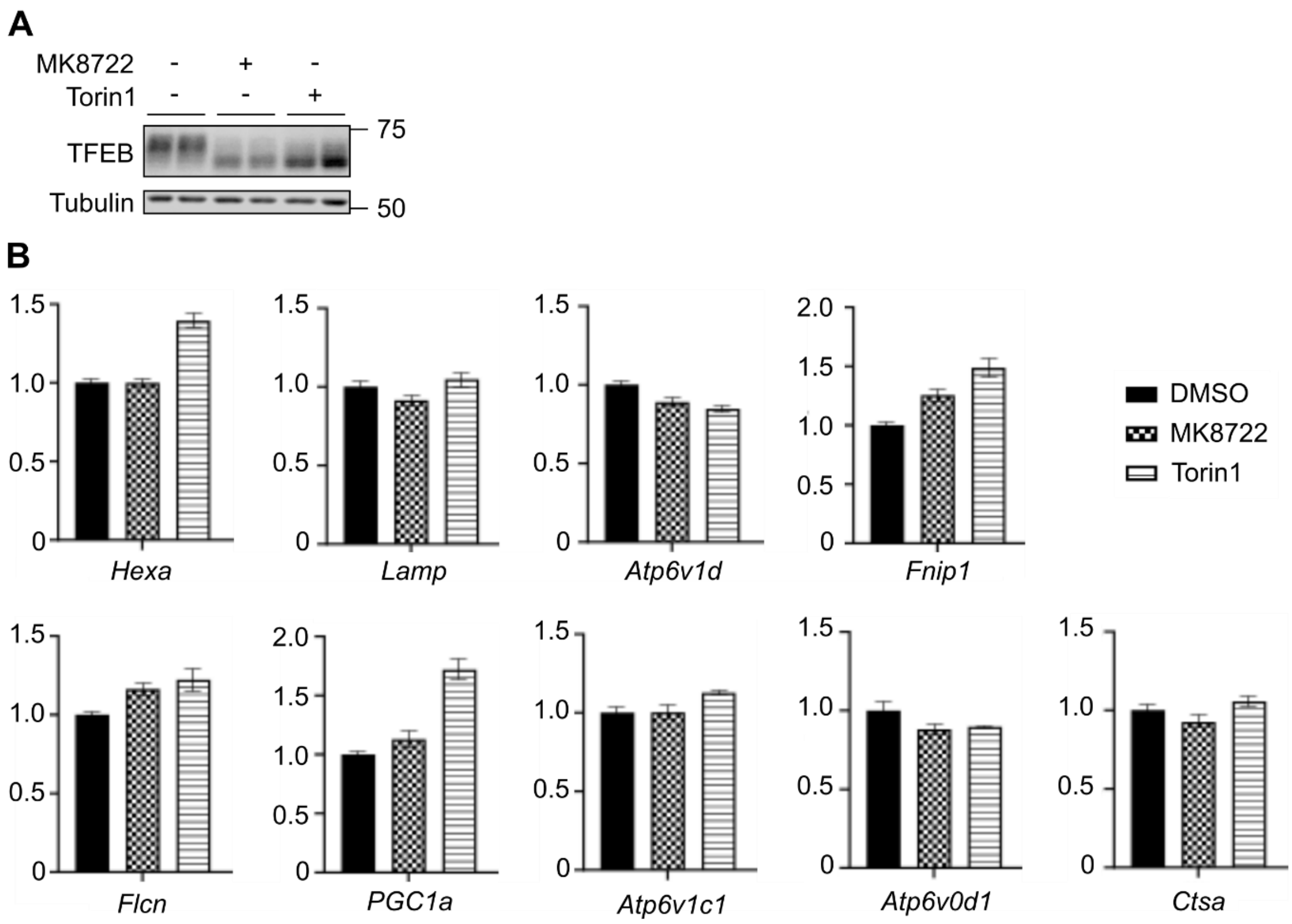

A
MK8722
Torin1
TFEB
Tubulin
75
50
B
Hexa
Lamp
Atp6v1d
Fnip1
Flcn
PGC1a
Atp6v1c1
Atp6v0d1
Ctsa
DMSO
MK8722
Torin1

Figure S1

Figure S1. TFEB dephosphorylation alone is insufficient to induce the expression of its target genes in HEK293 cells.

(A) HEK293 cells were treated with 10 μM MK8722 or 100 nM Torin1 for 6 hours. Cell lysates were subjected to western blot analysis using the indicated antibodies (N = 2 experiments). **(B)** HEK293 cells were treated with 10 μM MK8722 or 100 nM Torin1 for 6 hours. The relative mRNA levels of the indicated genes were measured by RT-qPCR. Data are representative of two independent experiments and are presented as mean ± SEM from N = 3 technical replicates.

Table S1. Primer sequences used for qPCR.

**Gene**	**Forward 5-3**	**Reverse 5-3**
Hexa	CAACCAACACATTCTTCTCCA	CGCTATCGTGACCTGCTTTT
Lamp	CGTGTCACGAAGGCGTTTTCAG	CTGTTCTCGTCCAGCAGACACT
Atp6v1d	ATAACCAACAGGCGTGTAAATGC	AGCCAGAAGATTAGCAGGCTC
Fnip1	GGTTCTCGGTGCTCTTCTGAT	GCTGTGGAGGGGAACGAAT
Flcn	GATTGAAGCGGCTCTGACCAAC	TCGACTGTCCACCTTGGTGAAC
PGC1a	CATGCAAATCACAATCACAGG	TTGTGGCTTTTGCTGTTGAC
Atp6v1c1	ATTGCATGCGGCAACTTCAA	CCAAGACATCCAACGTGCCA
Atp6v0d1	GAGGAACCATGCCTATGAGCC	GGATCACGTTGTCGATCATGTAA
Ctsa	CCAACACAACAGCTGCTTCC	CTGGGAGTTCATGCTTCGGT
Tbp	AGGGTTTCTGGTTTGCCAAGA	CTGAATAGGCTGTGGGGTCA
RNA18S	AACCCGTTGAACCCCATT	CCATCCAATCGGTAGTAGCG
Hprt	TGGCGTCGTGATTAGTGATG	AACACCCTTTCCAAATCCTCA
